# Socioeconomic decline and advancement within and between generations and the risk of stroke - a case-control study

**DOI:** 10.1186/s42466-019-0012-6

**Published:** 2019-03-15

**Authors:** Armin J. Grau, Annette Aigner, Christian Urbanek, Frederik Palm, Florian Buggle, Anton Safer, Heiko Becher

**Affiliations:** 1 Neurology Department, Klinikum Ludwigshafen a.Rh, Bremserstr.79, 67063 Ludwigshafen a.Rh, Germany; 2 Institute of Medical Biometry and Epidemiology, University Medical Hospital Hamburg-Eppendorf, Martinistraße 52, 20246 Hamburg, Germany; 3 Institute of Public Health, Charité – Universitätsmedizin Berlin, Charitéplatz 1, 10117 Berlin, Germany; 4 Department of Neurology, Klinikum Schleswig, St. Jürgener Str. 1-3, 24837 Schleswig, Germany; 5 Institute of Global Health, University of Heidelberg, INF 324, 69120 Heidelberg, Germany

**Keywords:** Socioeconomic conditions, Risk factor, Ischemic stroke

## Abstract

**Abstract:**

**Background:**

Disadvantageous socioeconomic conditions (SEC) in both childhood and adulthood increase the risk of stroke. We investigated whether intergenerational and lifetime social advancement decreases and/or social descent increases stroke risk.

**Methods:**

In a case-control study with 466 patients with first-ever ischemic stroke and 807 controls randomly selected from the general population, we compared paternal profession to subjects’ professional education in adolescence and their last profession in adulthood. Furthermore, we constructed a socioeconomic risk score for childhood (based on paternal and maternal profession and occupation, familial, living and material conditions), adolescence (based on highest school degree and professional education), and adulthood (based on last profession, periods of unemployment, and marital status), and compared subjects´ positions at different life stages. Odds ratios were derived based on conditional logistic regression conditioning on age and sex only, after adjustment for medical and lifestyle risk factors, and after additional adjustment for socioeconomic risk score values.

**Results:**

Intergenerational upward mobility between paternal profession and subject’s professional education was associated with lower ischemic stroke risk independent of medical and lifestyle risk factors (odds ratio (OR) 0.58; 95% confidence interval (CI) 0.41–0.81) and after additional adjustment for socioeconomic conditions in all three life stages (OR 0.67; 95% CI 0.45–0.99). Advancement between fathers´ profession and subject’s last profession was associated with reduced odds of stroke after adjustment for risk factors (OR 0.65; 95% CI 0.47–0.89), but not significantly after additional adjustment for SEC (OR 0.77; 95% CI 0.52–1.13). Social descent between adolescence and adulthood indicated by the transition into a more disadvantageous tertile of socioeconomic risk score was associated with increased odds of stroke after adjustment for all risk factor (OR 2.93; 95% CI 1.21–7.13). Analyses by sex revealed mostly similar results in men and women with only few potential differences.

**Conclusions:**

Our study results indicate that aspects of social downward mobility during adulthood may be associated with increased risk of stroke, whereas intergenerational upward mobility may be linked to a lower stroke risk. If confirmed by future studies, such results may help to focus stroke prevention measures at high risk populations.

## Background

Disadvantageous socioeconomic conditions are associated with increased incidence and mortality of stroke both in global perspectives and within populations worldwide [1, 2]. Low socioeconomic status is a risk factor for stroke that is associated with but not fully explained by common vascular risk factors [2]. Previously, we reported that disadvantageous socioeconomic conditions (SEC) in childhood are independently associated with higher risk of ischemic stroke later in adulthood [3]. Using a socioeconomic risk score, low socioeconomic conditions during both childhood (OR 1.77; 95% CI 1.20–2.60) and adulthood (OR 1.74; 95% CI 1.16–2.60) were associated with stroke risk after adjustment for common stroke risk factors and SEC in other life stages. For disadvantageous conditions during adolescence, we found a non-significant trend towards an association with stroke risk (OR 1.64; 95% CI 0.97–2.78). A recent meta-analysis confirms disadvantageous childhood SEC as a stroke risk factor [4]. Social upward mobility as compared to the parental generation and during own adulthood may be associated with higher self-consciousness and better health awareness and may thus, contribute to protection from vascular diseases and stroke. Downward mobility can lead to frustration and stressful life events that may increase stroke risk. However, ambitious behavior associated with social advancement may also pose stress to the individual and may thus increase stroke risk. Data on social mobility and risk of vascular diseases and particularly of stroke are scarce [5–10]. It is insufficiently understood whether social upward or downward mobility related to the parental generation or to social status in early adulthood would alter stroke risk. Therefore, we explored whether aspects of social advancement during lifetime are associated with reduced and aspects of social decline with increased risk of ischemic stroke.

### Subjects and methods

Within the Ludwigshafen Stroke Study (LuSSt), a population-based stroke registry [11], we performed a case-control study with patients with first-ever ischemic stroke (cases) and age- and sex-matched stroke-free controls, randomly selected from the general population [3]. LuSSt used standard definitions and multiple overlapping methods of case-ascertainment in order to identify all cases with incident stroke or transient ischemic attack among the population of Ludwigshafen am Rhein, a center of chemical industry with about 165,000 inhabitants.

The design of the case-control study was reported previously [3]. Shortly, inclusion criteria for cases and controls included age between 18 and 80 years, Caucasian ethnicity, permanent residency in the study area, and written informed consent to study participation. Inclusion criterion for cases was the diagnosis of a first-ever ischemic stroke based on an acute neurological deficit lasting > 24 h with no other reason than cerebral ischemia. All cases received neuroimaging (cerebral computed tomography or magnetic resonance imaging) excluding cerebral hemorrhage. For practical reasons, recruitment that took place from October 2007 to April 2012 was restricted to cases who were admitted to the Klinikum Ludwigshafen. Roughly 93% of all stroke patients under the age of 80 are admitted to the Neurology Department of the Klinikum Ludwigshafen which accommodates the only stroke unit within the city. Exclusion criteria for both cases and controls included any previous stroke, myocardial infarction within past 90 days, dementia, severe aphasia or any other relevant communication barrier, and severe disability which impeded participation in the interview.

Controls were recruited based on a random sample of Ludwigshafen residents drawn from the population registry. Selected subjects received invitation letters with detailed information on the study and an invitation for interview and examination to the Klinikum Ludwigshafen. Persons not responding were contacted by telephone or sent a reminder letter. Data collected included anthropometric measures, previous diseases, smoking, alcohol intake, physical activity, diet, medication, and socioeconomic history. Data were double entered and checked for completeness and plausibility.

We recruited 470 cases with first-ever ischemic stroke (188 women (40.0%); mean age 66.5 ± 10.8 years) and 282 men (mean age 65.5 ± 10.7 years)) and 809 control subjects (338 women (41.8%), mean age 66.4 ± 11.1 years; 471 men (58.2%), mean age 67.9 ± 9.5 years)). Participation rate was 73.7% in cases and 46.6% in controls [3].

Vascular risk factors and diseases were assessed as reported previously. [3] Information on SEC in childhood, adolescence, and adulthood was collected in detail and used to develop a socioeconomic risk score for each life period [3]. Risk score values for childhood were constructed based on paternal occupation (academic (0 points), non-academic white collar (1 point), blue collar and unskilled professions (2 points)), maternal occupation (academic and non-academic white collar professions, also including housewifes (0 points), blue collar and unskilled professions (1 point)), family conditions (growing-up with both parents (0 point), one parent (1 point), without parents (2 points)), number of siblings (0–3 (0 point), >3 (1 point), living conditions (rooms per person (> 1 (0 points), 0.5–1 (1 point), <0.5 (2 points); toilet in the house or apartment (yes (0 points), no (1 point)), parental car-ownership (yes (0 points), no (1 point)), estimated familial income as compared to class-mates (upper half (0 points), lower half (1 point)), and episodes of paternal unemployment (no (0 points), yes (1 point)). Risk score values ranged from 0 (advantageous conditions) to 12 (disadvantageous conditions). The risk score for adolescence was based on highest school degree (high school (12–13 school years, 0 points), middle school (10 school years, 2 points) and primary school (8–9 school years, 4 points)) and professional education (academic exam (0 points), non-academic white collar professional degree (1 point), skilled worker (2 points), unskilled worker including housewives (4 points)) and ranged from 0 to 8 points with higher numbers indicating lower educational levels. The risk score for adulthood was based on last profession before stroke or retirement (professions typically requiring academic training (0 points), non-academic white collar professions (1 point), skilled blue collar professions (2 points) and unskilled professions including housewives (4 points)), periods of unemployment > 6 months (no (0 points), yes (2 points)), and marital status (being married or living in partnership (0 points) versus being divorced, single or widowed (2 points)) [2]. The derived socioeconomic risk scores in childhood, adolescence and adulthood were each categorized into risk strata based on tertiles from controls´ values, thus representing low, middle and high risk strata. Social decline and advancement were defined as reaching any higher or lower tertile, respectively.

To examine occupational advancement or decline, we compared paternal professions with the first professional education in adolescence/early adulthood and subjects´ last profession (categorized as academic, white-collar non-academic (clerk), combined unskilled and skilled blue collar workers/other including housewives).

Approval by the ethics committee of the Landesärztekammer Rheinland-Pfalz (837.333.05(4991)) was obtained. All subjects gave written informed consent.

### Statistical analysis

We report absolute and relative frequencies of individual social mobility categories by case-control status and sex. Odds ratio (OR) estimates along with 95% confidence intervals (CI) are derived from conditional logistic regression. In a first model we only condition on 2-year-age and sex groups. A second model additionally adjusts for medical and lifestyle risk factors (hypertension, diabetes mellitus, hypercholesterolemia, atrial fibrillation, peripheral arterial disease, current smoking, frequent high alcohol consumption, low physical activity, low number of teeth and low number of dentist visits, cardiac failure, coronary heart disease, fruit and vegetable consumption). A third model additionally adjusted for socioeconomic risk scores in childhood, adolescence, and adulthood (model 3). This final model was also used for stratified analyses by sex. As all analyses are of exploratory nature, we did not adjust for multiple testing. Imputation of missing values was performed as reported previously [3]. Two cases and four controls yet had to be excluded**.** Data management was performed with the statistical software SAS, data analyses with R [12], conditional logistic regression with the R package survival [13], visualization with ggplot2 [14].

## Results

More control subjects than cases achieved a higher professional status as their fathers in both adolescence (30.1% (controls) versus 19.3% (cases)) and later adulthood (36.7% (controls) versus 23.6% (cases)). Professional advancement in adolescence as compared to paternal profession was associated with a reduced risk of stroke after adjustment for all stroke risk factors and socioeconomic status in childhood, adolescence, and later adulthood (OR 0.67; 95% CI 0.45–0.99). Professional advancement between fathers´ profession and subject’s last profession was associated with reduced odds of stroke after adjustment for medical and lifestyle risk factors (OR 0.65; 95% CI 0.47–0.89). About two thirds of stroke cases and about half of the controls belonged to the same professional category as their fathers in both adolescence (professional training; cases: 70.8%; controls: 57.0%) and late adulthood (last profession; cases: 67.0%; controls 52.4%). Thus, in the intergenerational comparison, consistency was the predominant pattern. Advancement between professional training (adolescence) and the last profession in late adulthood was not associated with stroke risk. Professional descent did not alter stroke risk in the multivariable models (Table 1).

**Table 1:** Professional status as measure of social mobility between childhood, adolescence and late adulthood

Variable	Category	Cases *n* = 466	Controls *n* = 807	Model variant		
				Model 1^a^	Model 2^b^	Model 3^c^
Fathers´ profession vs. subjects´ professional training	Advancement	90 (19.3%)	243 (30.1%)	0.50 (0.37–0.67)	0.58 (0.41–0.81)	0.67 (0.45–0.99)
	No change	330 (70.8%)	460 (57.0%)	1.00	1.00	1.00
	Descent	46 (9.9%)	104 (12.9%)	0.64 (0.43–0.95)	0.76 (0.48–1.20)	0.95 (0.59–1.53)
Fathers´ profession vs. subjects´ last profession	Advancement	110 (23.6%)	296 (36.7%)	0.52 (0.40–0.69)	0.65 (0.47–0.89)	0.77 (0.52–1.13)
	No change	312 (67.0%)	423 (52.4%)	1.00	1.00	1.00
	Descent	44 (9.4%)	88 (10.9%)	0.68 (0.45–1.02)	0.80 (0.50–1.28)	1.00 (0.61–1.63)
Professional training vs. last profession	Advancement	50 (10.7%)	68 (8.4%)	1.18 (0.79–1.78)	1.07 (0.67–1.72)	0.77 (0.44–1.34)
	No change	355 (76.2%)	607 (75.2%)	1.00	1.00	1.00
	Descent	61 (13.1%)	132 (16.4%)	0.87 (0.61–1.23)	0.96 (0.64–1.44)	0.95 (0.61–1.48)

*OR* odds ratio, *CI* confidence interval

^a^logistic regression model, conditioned on age (2-year age intervals) and sex

^**b**^ additionally adjusted for hypertension, diabetes mellitus, hypercholesterolemia, atrial fibrillation, coronary heart disease, peripheral arterial disease, cardiac failure, number of teeth, smoking, alcohol consumption, dentist visits, physical activity, fruit consumption, vegetable consumption

^**c**^ additionally adjusted for socioeconomic scores in childhood, adolescence, and adulthood [2]

In analyses of risk score tertiles, changes between childhood and both adolescence and later adulthood did not differ between cases and controls and were not associated with stroke risk. However, mobility into a higher risk score stratum between adolescence and later adulthood indicating social descent was linked to increased odds of stroke even in the fully adjusted model (OR 2.93; 95% CI 1.21–7.13) (Table 2).

**Table 2:** Social mobility between childhood, adolescence and late adulthood. Analysis of risk score strata

Variable	Category	Cases *n* = 466	Controls *n* = 807	Model variant		
				Model 1^a^	Model 2^b^	Model 3^c^
Childhood vs. adolescence	Advancement	130 (27.9%	211 (26.2%)	1.03 (0.78–1.37)	0.95 (0.68–1.31)	0.99 (0.45–2.22)
	No change	244 (52.4%)	406 (50.3%)	1.00	1.00	1.00
	Descent	92 (19.7%)	190 (23.5%)	0.84 (0.62–1.15)	0.77 (0.54–1.10)	0.64 (0.29–1.39)
Childhood vs. adulthood	Advancement	109 (23.4%)	208 (25.8%)	0.91 (0.67–1.24)	0.97 (0.68–1.39)	0.74 (0.39–1.41)
	No change	191 (41.0%)	339 (42.0%)	1.00	1.00	1.00
	Descent	166 (35.6%)	260 (32.2%)	1.07 (0.81–1.41)	1.02 (0.74–1.41)	1.21 (0.63–2.34)
Adolescence vs. adulthood	Advancement	59 (12.7%)	130 (16.1%)	1.01 (0.70–1.44)	1.27 (0.84–1.92)	0.58 (0.25–1.36)
	No change	235 (50.4%)	462 (57.3%)	1.00	1.00	1.00
	Descent	172 (36.9%)	215 (26.6%)	1.49 (1.14–1.95)	1.43 (1.05–1.95)	2.93 (1.21–7.13)

*OR* odds ratio, *CI* confidence interval

^a^ logistic regression model, conditioned on age (2-year age intervals) and sex

^**b**^ additionally adjusted for hypertension, diabetes mellitus, hypercholesterolemia, atrial fibrillation, coronary heart disease, peripheral arterial disease, cardiac failure, number of teeth, smoking, alcohol consumption, dentist visits, physical activity, fruit consumption, vegetable consumption

^**c**^ additionally adjusted for socioeconomic scores in childhood, adolescence, and adulthood [2]

**Fig. 1:**
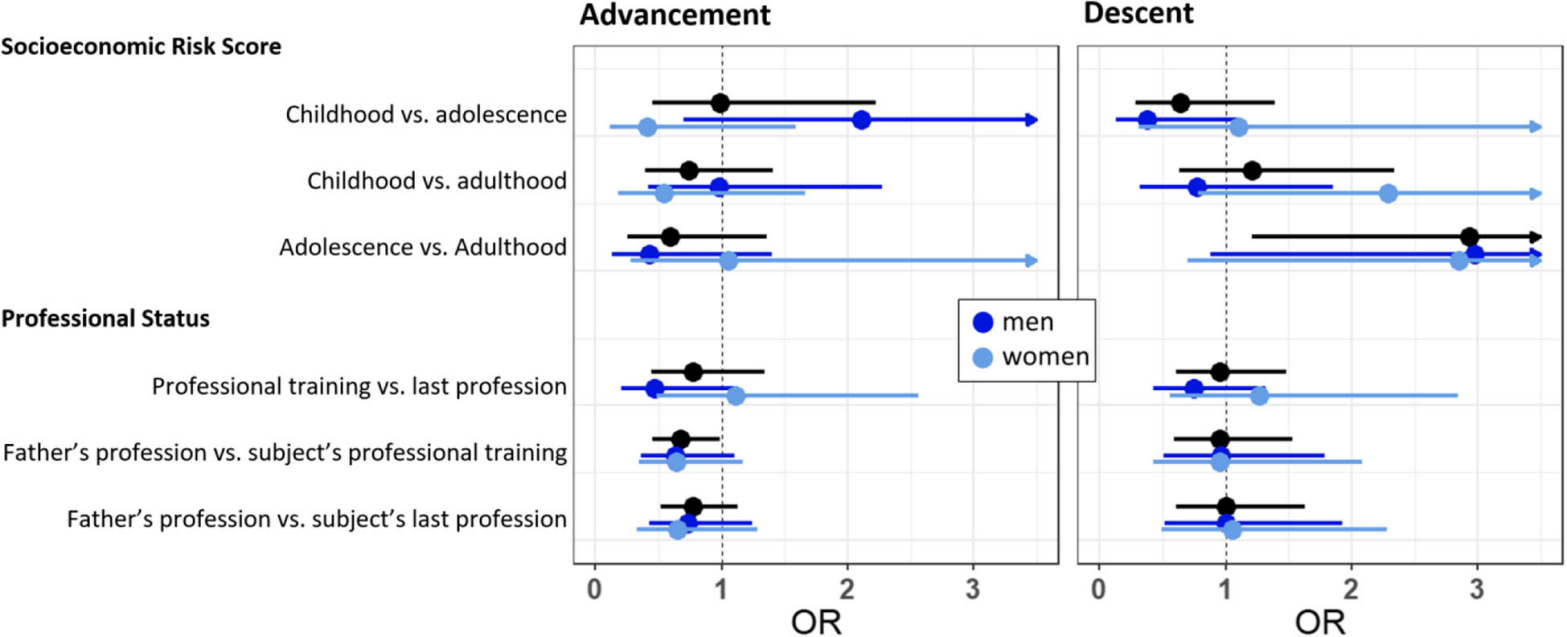
Adjusted as in Model 3 of Tables 1 and 2

Stratification by sex revealed mostly similar results in men and women with a few differences, especially for socioeconomic advancement when childhood and adolescence risk scores were compared. In this case advancement showed the tendency of being a risk factor for men but protective for women, although both effects were not significant (Fig. 1).

## Discussion

We assessed the association between social mobility and ischemic stroke risk using two different measures. Intergenerational professional advancement was associated with a lower risk of stroke. Using a multifaceted socioeconomic risk score at different life stages that did not only include professional status, relative socioeconomic downward mobility between adolescence and late adulthood was independently associated with increased stroke risk. This finding is not detected if only professional status was analyzed. Both results indicate that social advancement is rather associated with a lower risk and social decline with an increased risk of stroke.

Evidence that socioeconomic mobility may importantly influence cardiovascular risk is limited and data are scarce, particularly for stroke [5–10]. In Swedish women, moving from non-manual parental work to manual work in adulthood was linked to increased stroke mortality compared to stable non-manual occupation after adjustment for education [7]. Swedish women whose families were upwardly or downwardly mobile during their childhood had increased risk of ischemic stroke [8]. In a previous smaller case-control study, advancement from paternal manual work to non-manual work in the index person was associated with lower risk of stroke in univariable analysis but this did not reach significance after multivariable adjustment [9].

Social status inconsistency with lower educational attainment and higher occupational position was an independent risk factor of cardiovascular diseases including stroke in men, but not in women in a cohort study. Separate data on stroke were not reported [10]. In contrast to this finding, we did not detect any risk modification by differences between professional training and later professional status in men, however, a non-significant trend was seen in women.

Behavioral, psychological, and biological factors may contribute to the link between SEC including social mobility and stroke [6]. We adjusted for several behavioral factors, however, residual influences may still play a role. Social advancement may give rise to positive effects including health promoting behaviors and to an optimistic attitude although it can also pose psychological pressure to the individual. Downward mobility may adversely affect stroke risk through its impact on mental health. Perceived psychosocial stress, although being an imprecise term, is independently associated with the risk of stroke as is depression [6, 15].

There are several limitations of our study. Due to the inclusion and exclusion criteria of the study protocol we recruited mostly patients of German background. Thus, the role of migration could not be studied. Our study may also have been limited by a selection bias towards controls with a more active lifestyle and better chances of social advancement and by difficulties in assigning women to occupational groups in the same way as men. Our data are based on a case-control study; thus, causality cannot be inferred from the results. It is possible that less healthy subjects may be prone to both stroke and downward social mobility.

Despite such limitations, the results of our study are consistent and indicate that aspects of social downward mobility may be associated with increased risk and upward mobility may be linked to decreased risk of stroke. However, further studies and preferably larger and prospective ones are required to confirm our findings.

## Conclusions

The results from our study suggest that aspects of social downward mobility during adulthood may be linked to increased risk of stroke. In contrast, intergenerational upward mobility was associated with a lower stroke risk. If confirmed by future studies, such findings could assist in identifying high-risk populations and prevention programs should focus on socially underprivileged groups.
